# Supplementary material for: Low-Ammonium Environment Increases the Nutrient Exchange between Diatom–Diazotroph Association Cells and Facilitates Photosynthesis and N2 Fixation—a Mechanistic Modeling Analysis
Source: Cells. 2022 Sep 17;11(18):2911. doi: 10.3390/cells11182911 (PMC9497195; doi:10.3390/cells11182911)
Supplement: Supplementary file 1 [file cells-11-02911-s001.zip › cells-1878456-supplementary.pdf]

Supplementary material

Contents:

Supplementary text

Figure S1-S9

Tables S1

Supplementary text

No transfer NH_4^+ equation. In the main text, we mentioned that when NH_4^+ uptake equals the consumption of diatom, there is no transfer. We name the value of NH_4^+ concentration in this condition as the *no transfer NH_4^+concentration*. Here is the derivation of no transfer NH_4^+ concentration equation.

Firstly, we make the consumption of the diatom ($\mu \times Q_N^D$, only include N usage in growth) equal to NH_4^+ uptake ($V_{NH_4^+}$) and get Equation S1:

$$\mu \times Q_N^D = V_{NH_4^+} \qquad \text{Equation S1}$$

Then use Equation S2 to represent $V_{NH_4^+}$:

$$V_{NH_4^+} = V_{NH_4^+}^{max} \frac{[NH_4^+]}{[NH_4^+] + K_m} \qquad \text{Equation S2}$$

And then come to a new Equation S3:

$$\mu \times Q_N^D = V_{NH_4^+}^{max} \frac{[NH_4^+]}{[NH_4^+] + K_m} \qquad \text{Equation S3}$$

Finally, we only keep $[NH_4^+]$ on the left side and have Equation S4, which shows that *no transfer NH_4^+* can be only influenced by growth rate (μ), since all other parameters in the right side are constant.

$$[NH_4^+] = \frac{\mu \times Q_N^D \times V_{NH_4^+}^{max}/K_m}{1 - \mu \times Q_N^D/K_m} \qquad \text{Equation S4}$$

Derivation of the key equations. To help readers to better understand the key equations in the main text, in this section we describe the derivation of the key equations. The derivation used here is similar with a previous DDA modeling paper [1].

Figure S1 shows a schematic of the DDA model with flux notations. Here Q_i^j(pmol cell^{-1}) means the cellular quota of element *i* in *j*, and J_i^{jk} (pmol cell^{-1} d^{-1}) represents transport of *i* from *j* to *k*, where i = C or N and *j*,*k* = H (Heterocysts: Het), V (Vegetative cells: Veg) or D (Diatoms: Dia). In this derivation, we start with the dynamics of C and N quotas in three different cells.

Equation S5 and Equation S6 describe how C and N change in Het. Cellular C can be accumulated from Veg (J_C^{VH}) and can be consumed by N_2 fixation ($F_C^{N_2fix}$). Cellular N can be consumed by transferring to Veg ($-J_N^{HV}$) and can be obtained from N_2 fixation ($F_N^{N_2fix}$).

$$\frac{dQ_C^H}{dt} = J_C^{VH} - F_C^{N_2fix} \qquad \text{Equation S5}$$

$$\frac{dQ_N^H}{dt} = -J_N^{HV} + F_N^{N_2fix} \qquad \text{Equation S6}$$

Equation S7 and Equation S8 indicate element quotas dynamics ($\frac{dQ_C^V}{dt}$ and $\frac{dQ_N^V}{dt}$) in Veg. C can be obtained from photosynthesis in Veg (F_{pho}^V) and transferred from diatom (J_C^{DV}). And C can be consumed by being transferred to Het (J_C^{VH}) and biosynthesis (including growth and respiration) of Veg and Het ($\mu(Q_C^V + Q_C^H)(1+E)$). As for N, it can be obtained from Het and can be transferred to Dia. And it can also be consumed by biosynthesis of Veg and Het ($\mu(Q_N^V + Q_N^H)$).

$$\frac{dQ_C^V}{dt} = F_{pho}^V + J_C^{DV} - J_C^{VH} - \mu(Q_C^V + Q_C^H)(1+E) \qquad \text{Equation S7}$$

$$\frac{dQ_N^V}{dt} = J_N^{HV} - J_N^{VD} - \mu(Q_N^V + Q_N^H) \qquad \text{Equation S8}$$

Equation S9 and Equation S10 are cellular C and N dynamics ($\frac{dQ_C^D}{dt}$ and $\frac{dQ_N^D}{dt}$) in Dia. C can be obtained from photosynthesis in Dia (F_{pho}^D). And it can be consumed by transfer to Veg (J_C^{DV}) and biosynthesis (including growth and respiration) of Dia ($\mu Q_C^D(1+E)$). As for N, it can be obtained from Veg (J_N^{VD}) and from environmental NH_4^+ uptake ($V_{NH_4^+}$). And it can also be consumed by biosynthesis of Dia (μQ_N^D).

$$\frac{dQ_C^D}{dt} = F_{pho}^D - J_C^{DV} - \mu Q_C^D(1+E) \qquad \text{Equation S9}$$

$$\frac{dQ_N^D}{dt} = J_N^{VD} - \mu Q_N^D + V_{NH_4^+}$$ Equation S10

After we get all of the above equations, we assume a steady state, which means that all of the element dynamic terms ($\frac{dy}{dt}$) are zero. Then we sum up Equation S5, Equation S7 and Equation S9, and we get Equation S11, which is the C balance equation that we used in main text equation (1). And we also sum up Equation S6, Equation S8 and Equation S10, and get Equation S12, which is the N balance equation that we used in main text equation (3).

$$F_{pho}^D + F_{pho}^V = \mu(Q_C^V + Q_C^H + Q_C^D)(1 + E) + F_C^{N_2fix}$$ Equation S11: the same as Equation (1) in the main text

$$F_N^{N_2fix} + V_{NH_4^+} = \mu(Q_N^V + Q_N^H + Q_N^D)$$ Equation S12: the same as Equation (3) in the main text

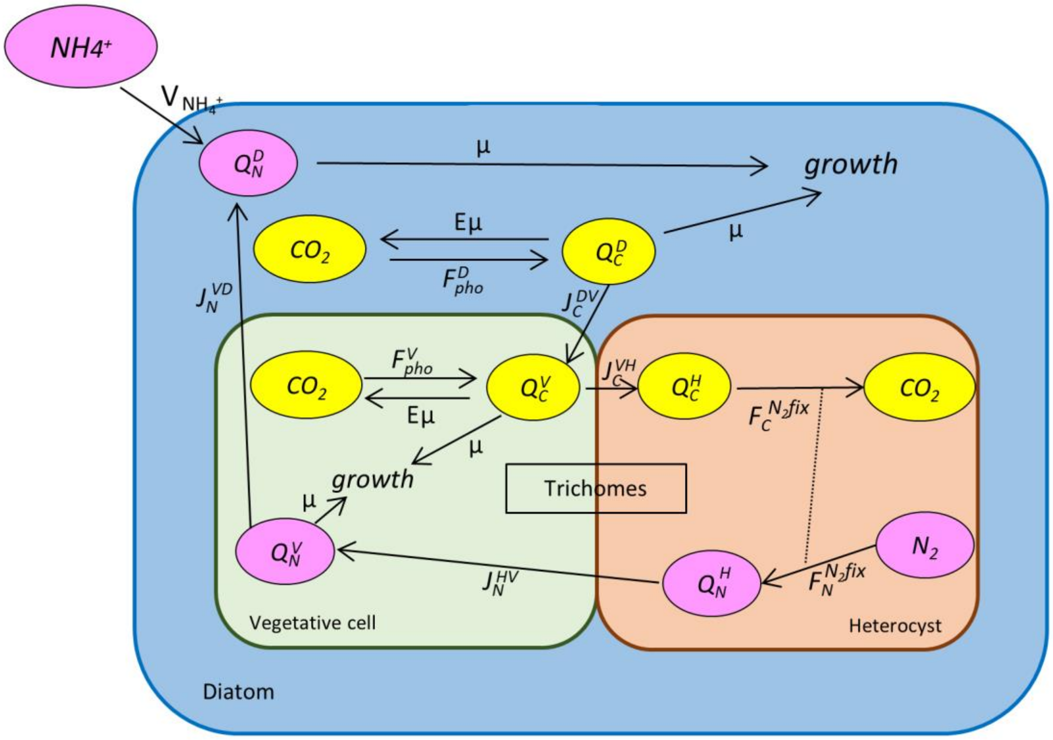

Figure S1. A schematic of the DDA model with flux notations. Blue frame and space: the host diatom. Green space and green frame: Vegetative cell in trichomes. Brown frame and light brown space: Heterocyst in trichomes. Yellow ovals: C pools. Pink ovals: N pools. Black arrows: The direction of element flux when the NH_4^+ supply is not

enough for N consumption in the host diatom cell. Red arrows: Direction change when NH_4^+ supply is more than the diatom consumption. Dash line: these are coupling between the processes. See Supplementary Methods for flux notations. Eμ represents E × μ, growth-dependent respiration.

Light intensity. Here we describe the equation (Equation S13) using light intensity as a variable in the calculation of photosynthesis. We assumed that total photosynthesis rate ($F_{pho}^{D} + F_{pho}^{V}$) can increase with light intensity (I) but becomes saturated when it reaches a maximum (F_{Cfix}^{max}, this is the maximum photosynthesis rate for the whole DDA). Here, A_i represents the light saturation coefficient. We got F_{Cfix}^{max} and A_i values from a previous modeling paper [2]. Given DDAs are often observed near the surface, we assumed strong enough light to allow saturation for both diatom and vegetative cells. And we also tested 50% maximum photosynthesis for vegetative cells to see if the difference of light-harvesting systems between diatom and cyanobacteria can change the results.

$$F_{pho}^{D} + F_{pho}^{V} = F_{Cfix}^{max}(1 - e^{-A_i I}) \qquad \text{Equation S13}$$

The simulation results of light effect are shown in Figure S1. Here, we found that light intensity can only influence C pathways (Figure S2). With increasing light intensity, photosynthesis rate and C transfer increase (Figure S2 a, c, e, f). However, N pathways are not influenced by light intensity (Figure S2 b, d). And the light effect is nearly identical when we tested 50% maximum photosynthesis for vegetative cells (Figure S3).

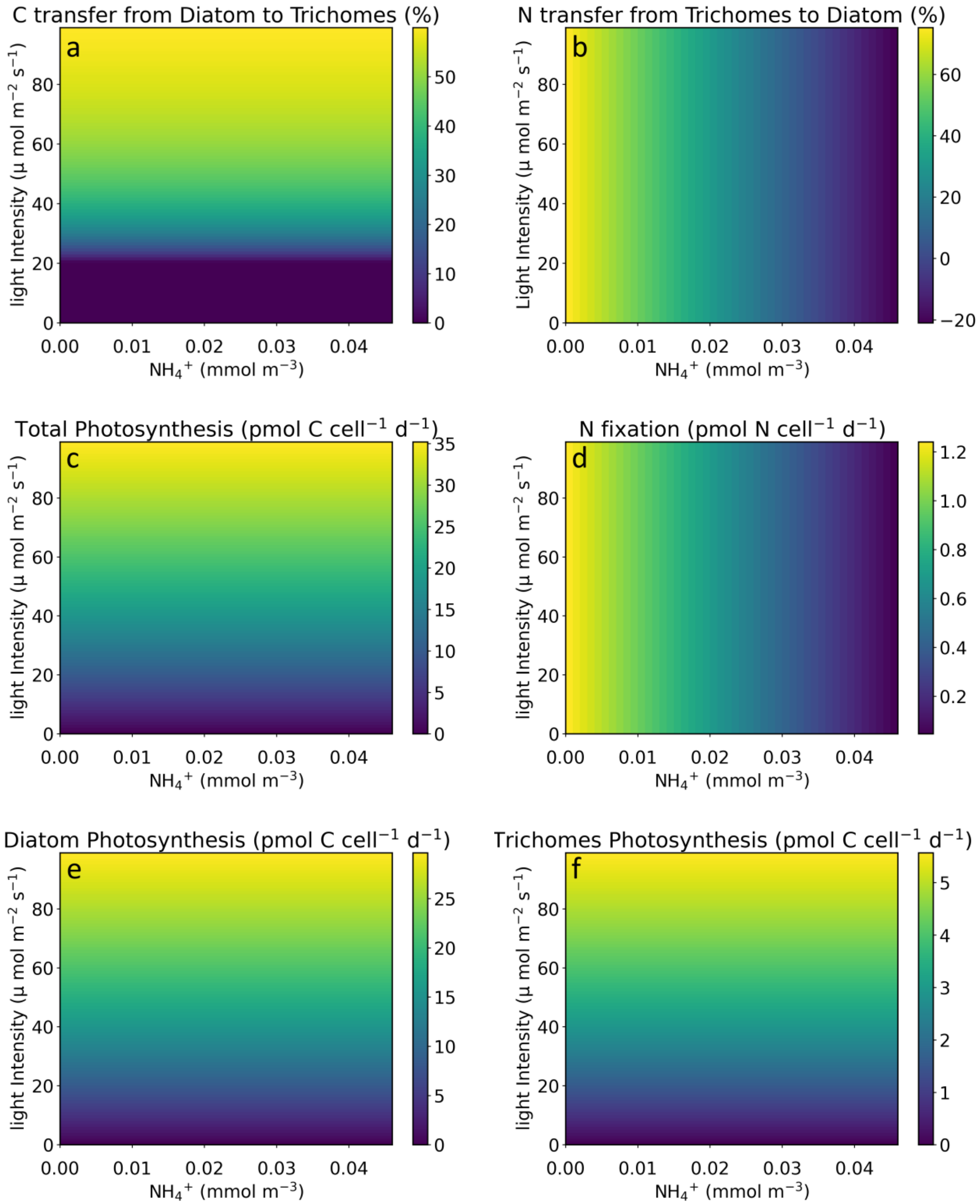

Figure S2. Simulated influence of the NH_4^+ concentration and light intensity on element transfer and supply. (a) Effect of NH_4^+ and light intensity on C transfer. (b) Effect of NH_4^+ and light intensity on N transfer. (c), (e), (f)

Effect of NH_4^+ and light intensity on photosynthesis, including total DDA, diatom and trichomes. (d) Effect of NH_4^+ and light intensity on N_2 fixation. For (a) and (b), the unit percentage means how much C and N transfer account for the total C and N consumption (or supply).

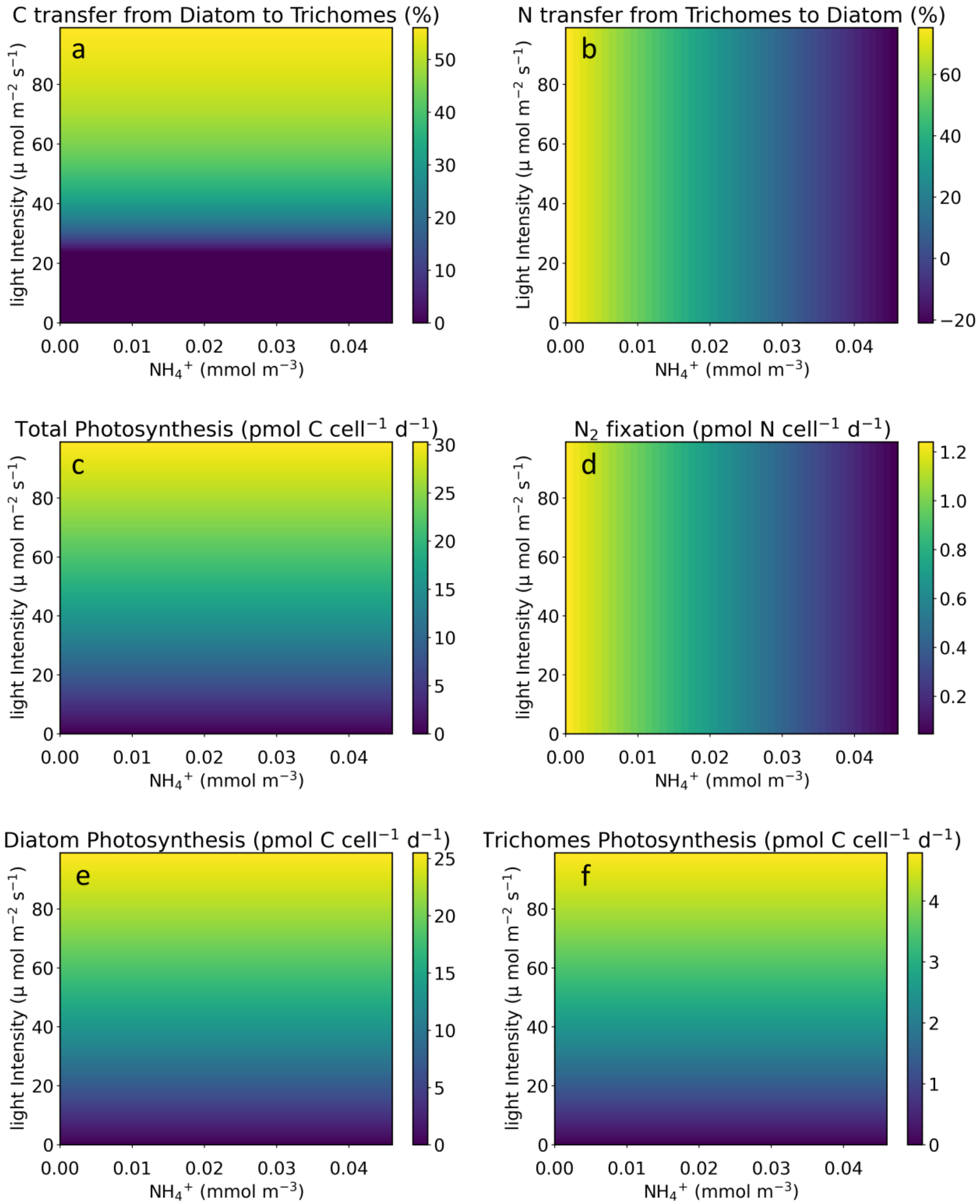

Figure S3. Sensitivity test: Simulated influence of the NH_4^+ concentration and light intensity on element transfer and supply (50% maximum photosynthesis for vegetative cells). (a) Effect of NH_4^+ and light intensity on C transfer. (b) Effect of NH_4^+ and light intensity on N transfer. (c), (e), (f) Effect of NH_4^+ and light intensity on photosynthesis,

including total DDA, diatom and trichomes. (d) Effect of NH_4^+ and light intensity on N_2 fixation. For (a) and (b), the unit percentage means how much C and N transfer account for the total C and N consumption (or supply).

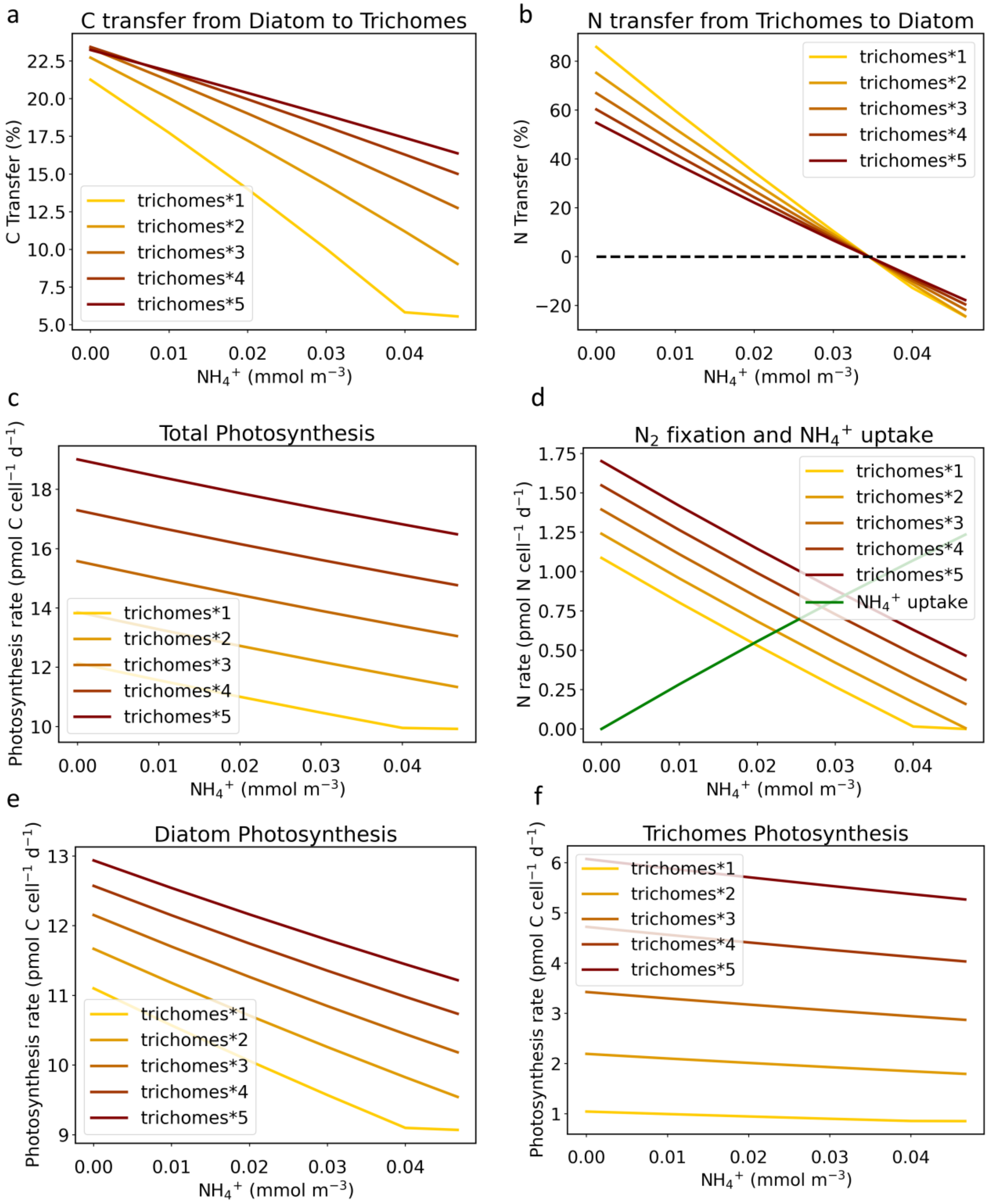

Figure S4. Simulated influences of the NH_4^+ concentration and trichomes numbers on element transfer and supply. (a) Effect of NH_4^+ and trichomes number on C transfer. (b) Effect of NH_4^+ and trichomes numbers on N transfer, the dash line is the no transfer NH_4^+ concentration. (c), (e), (f) Effect of NH_4^+ and trichomes numbers on photosynthesis, including total DDA, diatom and trichomes. (d) Effect of NH_4^+ and trichomes number on NH_4^+ uptake and N_2 fixation. The different color lines mean different number of trichomes. For (a) and (b), the unit percentage means how much C and N transfer account for the total C and N consumption (or supply).

Trichomes numbers. Here, we showed the effect of the number of trichomes on C and N transfer, photosynthesis and N_2 fixation.

Growth rate.

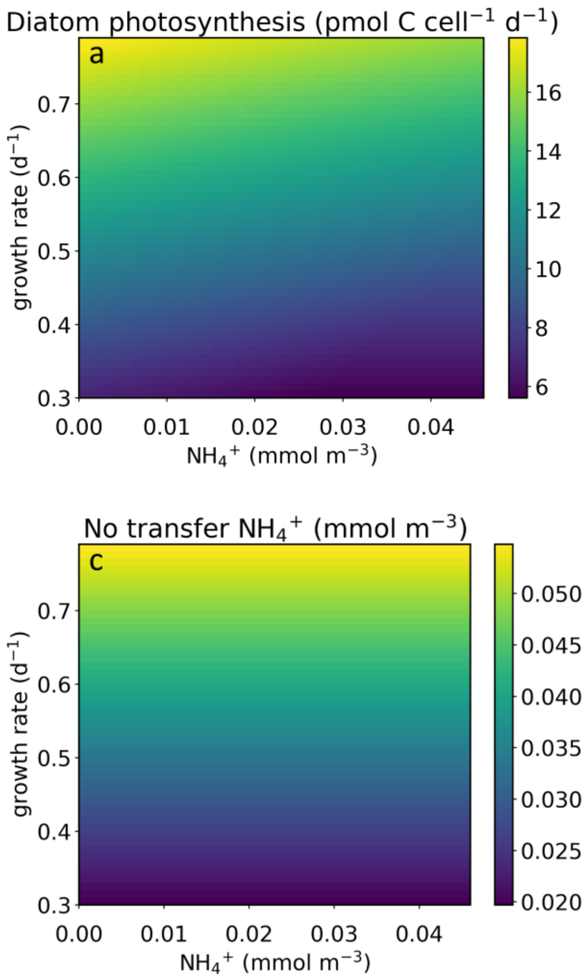

Figure S5. Simulated influences of the NH_4^+ concentration and growth rate on photosynthesis and the no transfer NH_4^+. These are supplementary figures for the main text growth rate figures (main text Figure 3).

Parameter table

Table S1. Parameters, units, and definitions for all equations

Parameter	Unit	Definition
F_{pho}^{D}	pmol C d^{-1} cell^{-1}	Diatom photosynthesis rate

F_{pho}^{V}	pmol C d^{-1} cell^{-1}	Vegetative cells photosynthesis rate
μ	d^{-1}	DDA growth rate
Q_{C}^{V}	pmol C d^{-1} cell^{-1}	cellular C quotas of vegetative cells per DDA
Q_{C}^{H}	pmol C d^{-1} cell^{-1}	cellular C quotas of heterocysts per DDA
Q_{C}^{D}	pmol C d^{-1} cell^{-1}	cellular C quotas of diatom per DDA
E	dimensionless unit	The ratio of respiration to biosynthesis
$F_{C}^{N_2fix}$	pmol C d^{-1} cell^{-1}	C usage in N_2 fixation
$F_{N}^{N_2fix}$	pmol N d^{-1} cell^{-1}	N usage in N_2 fixation
$F_{C:N}^{N_2fix}$	pmol C pmol N^{-1}	C to N cost ratio in N_2 fixation
$V_{NH_4^+}$	pmol N d^{-1} cell^{-1}	Diatom NH_4^+ uptake rate
Q_{N}^{V}	pmol N d^{-1} cell^{-1}	cellular N quotas of vegetative cells per DDA
Q_{N}^{H}	pmol N d^{-1} cell^{-1}	cellular N quotas of heterocysts per DDA
Q_{N}^{D}	pmol N d^{-1} cell^{-1}	cellular N quotas of diatom per DDA
$[NH_4^+]$	mmol m^{-3}	NH_4^+ concentration

$V_{NH_4^+}^{max}$	pmol N d^{-1} $cell^{-1}$	maximum NH_4^+ uptake rate
K_m	mmol m^{-3}	Half saturation concentration
F_{Cfix}^{max}	pmol C d^{-1} $cell^{-1}$	Maximum photosynthesis rate
A_i	μ mol^{-1} m^2 s	Light saturation coefficient
I	μ mol m^{-2} s^{-1}	Light intensity
$\frac{dQ_C^H}{dt}$	pmol C d^{-1} $cell^{-1}$	Cellular C dynamics in heterocysts
$\frac{dQ_N^H}{dt}$	pmol N d^{-1} $cell^{-1}$	Cellular N dynamics in heterocysts
$\frac{dQ_C^V}{dt}$	pmol C d^{-1} $cell^{-1}$	Cellular C dynamics in vegetative cells
$\frac{dQ_N^V}{dt}$	pmol N d^{-1} $cell^{-1}$	Cellular N dynamics in vegetative cells
$\frac{dQ_C^D}{dt}$	pmol C d^{-1} $cell^{-1}$	Cellular C dynamics in diatoms
$\frac{dQ_N^D}{dt}$	pmol N d^{-1} $cell^{-1}$	Cellular N dynamics in diatoms
J_C^{VH}	pmol C d^{-1} $cell^{-1}$	C transfer from vegetative cells to heterocysts
J_N^{HV}	pmol N d^{-1} $cell^{-1}$	N transfer from heterocysts to vegetative cells
J_C^{DV}	pmol C d^{-1} $cell^{-1}$	C transfer from diatoms to vegetative cells

J_N^{VD}	pmol N d^{-1} $cell^{-1}$	N transfer from vegetative cells to diatoms

Sensitivity test for changing the respiration to biosynthesis value

In our model, we assume that the ratio of respiration to biosynthesis (E) is constant for diatoms, heterocysts, and vegetative cells. To make our conclusions more convincing, we also did a sensitivity test to explore whether our results would be different if we used different values for the three types of cells. We used the original E value (0.38) for diatoms and heterocysts, and doubled the E value for vegetative cells. The results are in Figure *S6*, Figure *S7*, Figure *S8*, Figure *S9*. Comparing the results in these figures: Figure *S6* and main text Figure 2, Figure *S7* and main text Figure 3, Figure *S8* and Figure *S4*, we believe that the results are almost the same. And compared to main text Figure 5, Figure *S9* shows higher C transfers, but the conclusion is still the same. In conclusion, this sensitivity test shows that using different E values does not influence the overall conclusion from the simulation results. Therefore, using the same value is fine.

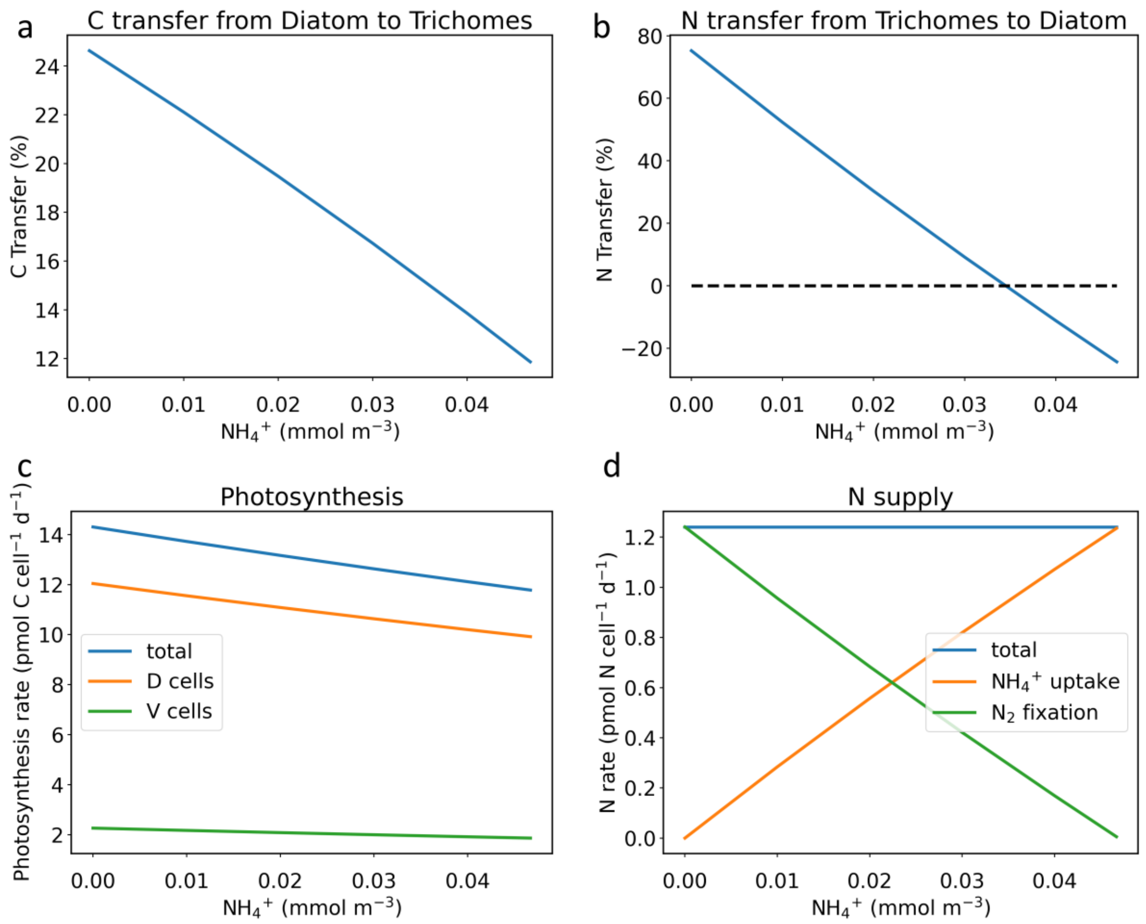

Figure S6. Sensitivity test: Simulated influences of the NH_4^+ concentration on element supply and transfer. (a) Effect of NH_4^+ on C transfer. (b) Effect of NH_4^+ on N transfer, the dash line is *no transfer NH_4^+ concentration*. (c) Effect of NH_4^+ on photosynthesis, blue line is photosynthesis change in DDA, orange line is photosynthesis change in the host diatom, and green line is photosynthesis change in vegetative cell. (d) Effect of NH_4^+ on N supply, blue line is total N supply change, orange line is NH_4^+ uptake change, and green line is N_2 fixation change. For (a) and (b), the unit percentage means how much C and N transfer account for the total C and N consumption (or supply).

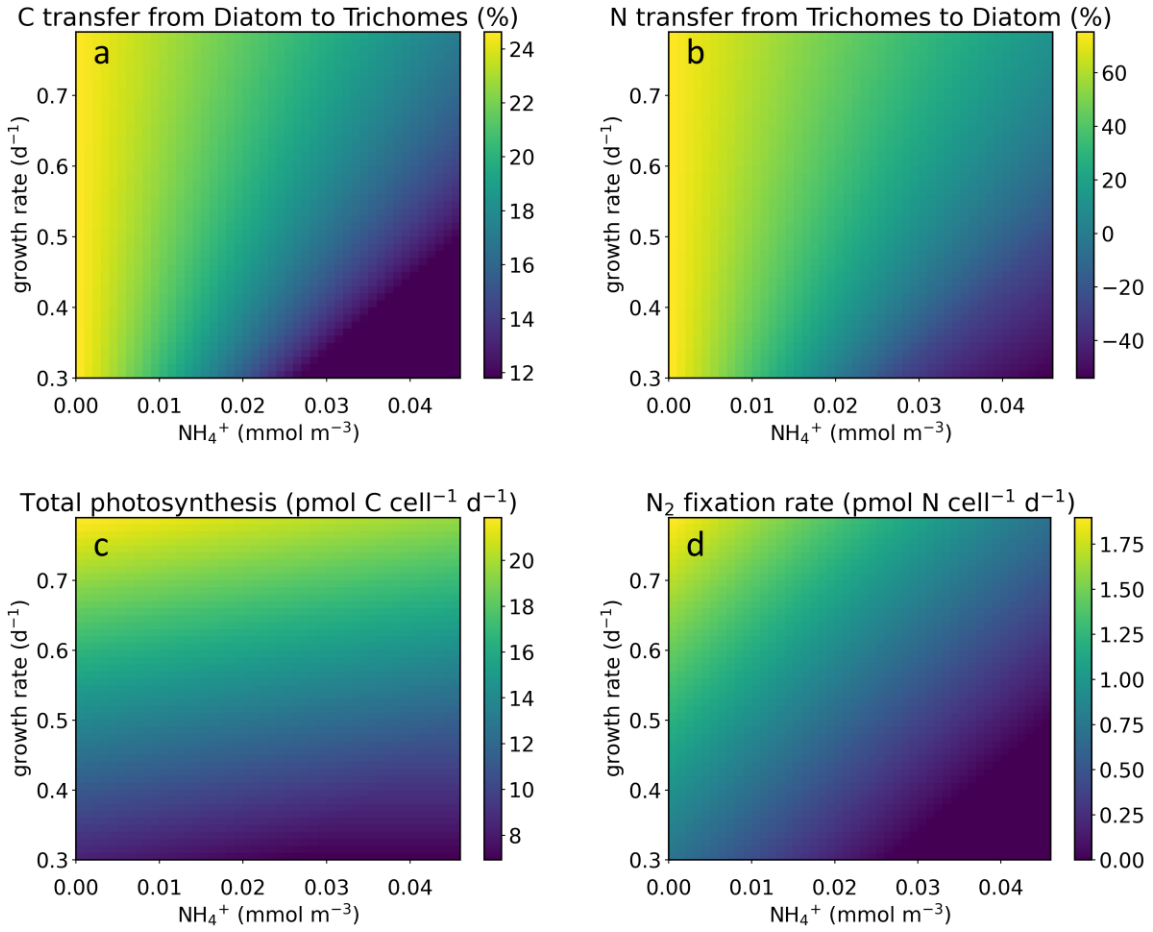

Figure S7. Sensitivity test: Simulated influence of the NH_4^+ concentration and growth rate on element transfer and supply. (a) Effect of NH_4^+ and growth rate on C transfer. (b) Effect of NH_4^+ and growth rate on N transfer. (c) Effect of NH_4^+ and growth rate on photosynthesis. (d) of NH_4^+ and growth rate on N_2 fixation. For (a) and (b), the unit percentage means how much C and N transfer account for the total C and N consumption (or supply).

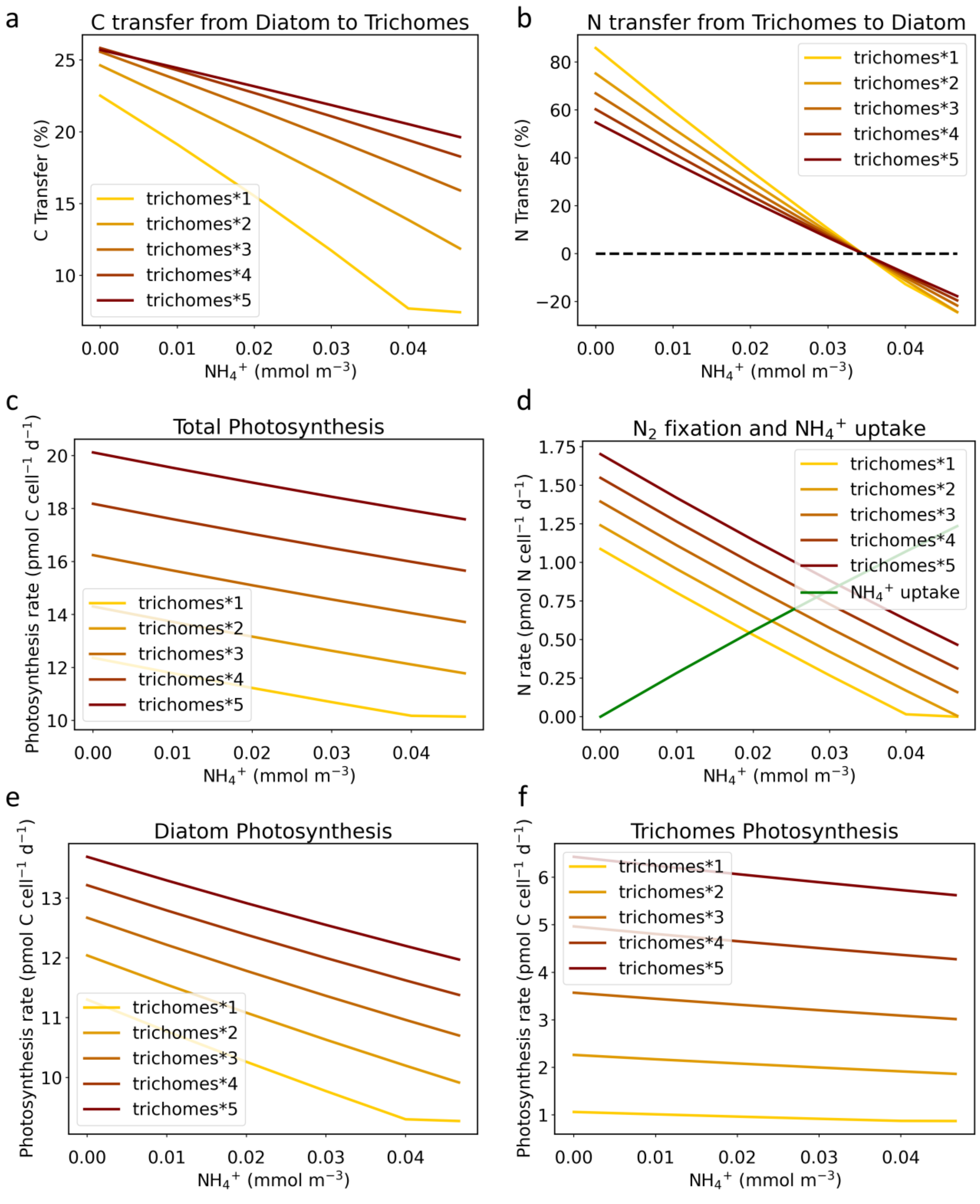

Figure S8. Sensitivity test: Simulated influences of the NH_4^+ concentration and trichomes numbers on element transfer and supply. (a) Effect of NH_4^+ and trichomes number on C transfer. (b) Effect of NH_4^+ and trichomes numbers on N transfer, the dash line is no transfer NH_4^+ concentration. (c), (e), (f) Effect of NH_4^+ and trichomes numbers on photosynthesis, including total DDA, diatom and trichomes. (d) Effect of NH_4^+ and trichomes number

on NH_4^+ uptake and N_2 fixation. The different color lines mean different number of trichomes. For (a) and (b), the unit percentage means how much C and N transfer account for the total C and N consumption (or supply).

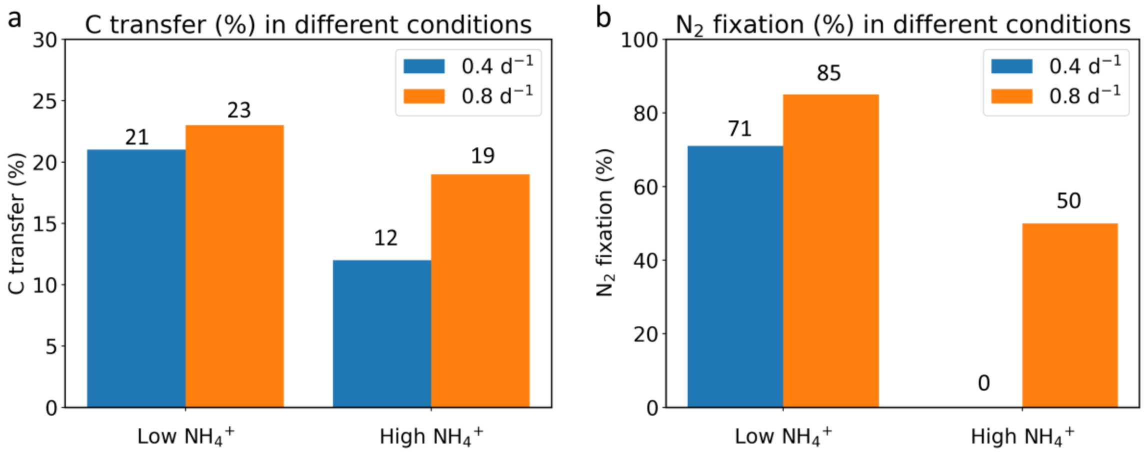

Figure S9. Sensitivity test: C transfer and N_2 fixation in different conditions: low NH_4^+, high NH_4^+, low growth rate (blue bars), high growth rate (orange bars). (a) C transfer. (b) N_2 fixation.

References

1. Inomura, K.; Follett, C.L.; Masuda, T.; Eichner, M.; Prášil, O.; Deutsch, C. Carbon Transfer from the Host Diatom Enables Fast Growth and High Rate of N_2 Fixation by Symbiotic Heterocystous Cyanobacteria. *Plants* **2020**, *9*, 8–16, doi:10.3390/plants9020192.

2. Inomura, K.; Wilson, S.T.; Deutsch, C. Mechanistic Model for the Coexistence of Nitrogen Fixation and Photosynthesis in Marine Trichodesmium. *mSystems* **2019**, *4*, 1–13.
